# Mindfulness-based cognitive therapy for inflammatory bowel disease patients: findings from an exploratory pilot randomised controlled trial

**DOI:** 10.1186/s13063-015-0909-5

**Published:** 2015-08-25

**Authors:** Mariyana Schoultz, Iain Atherton, Angus Watson

**Affiliations:** Centre for Health Science, School of Health Sciences, University of Stirling, Inverness, Scotland UK; Nursing, Midwifery & Social Care, Napier University, Edinburgh, Scotland UK; Raigmore Hospital NHS Highland, Inverness, Scotland UK

**Keywords:** Mindfulness-based cognitive therapy, MBCT, Inflammatory bowel disease, Crohn’s disease, Ulcerative colitis, Depression, Anxiety, Quality of life, Pilot randomised controlled trial

## Abstract

**Background:**

Inflammatory bowel disease (IBD) is a chronic gastrointestinal condition with a relapsing disease course. Managing the relapsing nature of the disease causes daily stress for IBD patients; thus, IBD patients report higher rates of depression and anxiety than the general population.

Mindfulness-based Cognitive Therapy (MBCT) is an evidence-based psychological program designed to help manage depressive and stress symptoms. There has been no randomized controlled trial (RCT) testing the use of MBCT in IBD patients.

The purpose of this pilot study is to test the trial methodology and assess the feasibility of conducting a large RCT testing the effectiveness of MBCT in IBD.

**Methods:**

The IBD patients, who were recruited from gastroenterology outpatient clinics at two Scottish NHS Boards, were randomly allocated to an MBCT intervention group (n = 22) or a wait-list control group (n = 22). The MBCT intervention consisted of 16 hours of structured group training over 8 consecutive weeks plus guided home practice and follow-up sessions. The wait-list group received a leaflet entitled ‘Staying well with IBD’. All participants completed a baseline, post-intervention and 6-month follow up assessment. The key objectives were to assess patient eligibility and recruitment/dropout rate, to calculate initial estimates of parameters to the proposed outcome measures (depression, anxiety, disease activity, dispositional mindfulness and quality of life) and to estimate sample size for a future large RCT.

**Results:**

In total, 350 patients were assessed for eligibility. Of these, 44 eligible patients consented to participate. The recruitment rate was 15 %, with main reasons for ineligibility indicated as follows: non-response to invitation, active disease symptoms, planned surgery or incompatibility with group schedule. There was a higher than expected dropout rate of 44 %. Initial estimates of parameters to the proposed outcomes at post-intervention and follow-up showed a significant improvement of scores in the MBCT group when compared to the control for depression, trait anxiety and dispositional mindfulness. The sample-size calculation was guided by estimates of clinically important effects in depression scores.

**Conclusions:**

This pilot study suggests that a multicentre randomized clinical trial testing the effectiveness of MBCT for IBD patients is feasible with some changes to the protocol. Improvement in depression, trait anxiety and dispositional mindfulness scores are promising when coupled with patients reporting a perceived improvement of their quality of life.

**Trial registration:**

ISRCTN27934462. 2 August 2013.

**Electronic supplementary material:**

The online version of this article (doi:10.1186/s13063-015-0909-5) contains supplementary material, which is available to authorized users.

## Background

### Impact of physical symptoms

Inflammatory bowel disease (IBD) is a group of idiopathic, chronic and disabling gastrointestinal conditions with a relapsing disease course. The two main types are Crohn’s disease (CD) and ulcerative colitis (UC), both characterised by symptomatic periods (flare-ups) combined with less-symptomatic periods (remission) [1]. IBD symptoms are caused by inflammation of the intestinal mucosa (the lining of the gut), and the most common symptoms are bloody diarrhoea, vomiting, severe pain and malnutrition [2, 3].

Managing and learning to cope with the relapsing nature of the disease causes daily stress for IBD patients. As a result, high rates of IBD patients report anxiety and depression not only when in symptomatic periods, but even in remission [4, 5]. The prolonged effects of pain, anxiety and depression have damaging effects on psychosocial functioning and quality of life (QoL) [6]. Poor quality of life is further associated with symptom relapse [7, 8]. Thus, anxiety, depression and relapse appear to be concomitant in a self-perpetuating cycle with devastating effects for IBD patients.

### Current management and limitations

Medication is the first line of treatment for patients with IBD. The therapeutic goal is to induce disease remission and keep symptoms at bay for as long as possible [9]. In addition, antidepressants are used for reducing distress, anxiety and depression [10–12]. However, the medication approach on its own is not without limitations. Firstly, it is reported that up to 40 % of IBD patients regularly omit their prescribed medications with a third of IBD patients still developing flare-ups even when complying with prescribed medication [13, 14]. Further to this, those using antidepressants often report unpleasant side effects while others report that antidepressants have no effect on their low mood or anxiety [15–17].

These limitations are a cause for concern and have prompted researchers and clinicians to look at other possible ways of symptom management and improving psychosocial functioning.

Accordingly, an alternative evidence-based therapeutic approaches focusing on stress management could have the potential to manage disease flare-ups and ultimately improve overall QoL [18–21].

### Mindfulness-based cognitive therapy

Mindfulness-based cognitive therapy (MBCT) is an evidence-based psychological group program designed to help manage stress and depressive symptoms [22, 23]. The core skill taught in the program is mindfulness, which is developing a non-judgemental awareness of one’s own thoughts, emotions, body sensations and their interactions. The mindfulness skill is taught via a series of meditation practices, cognitive behavioural exercises and discussions [24]. The MBCT program curriculum is structured and delivered over 8 weeks in a group setting. Through practicing the curriculum exercises in the group and at home, participants gradually develop better awareness and understanding of their individual responses to stress (psychological or physical) and learn new alternative ways to respond to stress. The evidence suggests that at program completion, participants would experience reduced negative effects from pain, distress, anxiety and depressive symptoms [25].

The clinical effectiveness of mindfulness-based therapies is evident in chronic pain conditions [26] and chronic medical conditions [27] such as fibromyalgia, cardiac and cancer patients, tinnitus and chronic fatigue syndrome [28, 29]. Mindfulness-based intervention has an anti-inflammatory effect on pro-inflammatory cytokine profiles in patients with prostate and breast cancer [30]. Systematic review and a meta-analysis of the effectiveness of mindfulness-based interventions on anxiety, depression and psychological distress in patients with chronic conditions have shown positive effects [31, 32]. Hence, the National Institute for Health and Care Excellence guidelines recommends the MBCT program as a preferred psychological therapy in the ‘clinical management of persistent sub-threshold depressive symptoms or mild, moderate or severe depression in adults (including people with a chronic physical health problem)’ [33].

A recent RCT suggests that mindfulness-based therapy has some benefit on IBD patients with IBS-like symptoms [34] and mindfulness-based therapy might be useful for UC patients with high stress reactivity [35]. However, MBCT and its effect on depression, anxiety and QoL have never been researched in a RCT with both Crohn’s and ulcerative colitis patients.

Due to the literature gap, and in accordance with the MRC guidance for development and evaluation of complex interventions [36], the aim of this study was to pilot the mindfulness-based cognitive therapy (MBCT) program with inflammatory bowel disease (IBD) patients and to evaluate the feasibility of conducting a full-scale RCT that will test the effectiveness of MBCT for IBD patients. The specific objectives were as follows:

1. Objective one was to assess eligibility and recruitment/dropout rate.
2. Objective two was to obtain initial estimates on parameters of the proposed outcome measures (depression, anxiety, quality of life, mindfulness and disease activity).
3. Objective three was to estimate a sample size for a large scale RCT.

## Methods

### Design and ethics

This study was a two-centre, two-arm, exploratory pilot RCT (MBCT treatment versus wait-list control group) with three assessments (baseline, post-treatment and 6 months). The full protocol of the study reported in this paper is the phase 1 of a two-phase pilot RCT described elsewhere [37]. Phase 2 will be reported separately. There were no deviations from the previously described protocol. All pilot data were collected between April 2013 and March 2014. The study was approved by the North Research Ethics Committee for North of Scotland on 8 April 2013 (REC ref 13/NF/0018). NHS Highland and NHS Grampian R&D Management Approval was obtained on 9 April 2013 and 14 September 2013, respectively. The trial was registered on the ISRCTN register (ISRCTN27934462) on 02 August 2013.

### Setting and recruitment

The study took place across two national health boards in the north of Scotland, a broad geographical area comprising urban and remote rural locations with approximate population of 800,000 people. Recruitment focused on outpatient gastroenterology clinics in the two areas.

Between May and October 2013, clinical staff at participating gastroenterology outpatient clinics scanned and identified potential participants that met the study inclusion criteria. Then, either study invitation packs were sent to patients with researchers contact details or patients seen consecutively in clinics were approached with the study information. All study information was co-designed with patients from the patient-involvement group [38]. Interested participants then registered their interest with the researcher by telephone or email. This was followed up with a screening visit with the researcher and then informed written consent was obtained. The inclusion criteria were broad enough to allow the sample to be representative of those diagnosed with IBD. Patients were excluded if they had a major psychiatric illness or alcohol dependency, were scheduled for surgery during the study period; if they were participating in other pharmacological or psychological intervention study or had a recent change of antidepressants or exacerbated symptoms. A full list of inclusion and exclusion criteria is in Additional file 1.

### Randomisation

Randomisation was performed after all participants had given written consent and baseline data had been collected. Participants were randomly allocated to the intervention ‘MBCT group’ or ‘wait-list control group’ in a 1:1 ratio. To ensure similarity between the groups, randomization was stratified on two variables - disease type and sex. Random allocation was computer generated. A permuted block randomization procedure with randomly varied block sizes was used. Blinding of researchers and patients was not possible because the intervention involved attending a course. Participants were informed of the results of randomization by email or letter (depending on their preference).

### Minimising bias

Bias can occur at any stage of planning, data collection, analysis or publication [39]. The following steps were taken to minimise systematic errors or bias and improve rigour: all participants self-completed all of the questionnaires, data entry was done by the lead researcher and was independently checked by a second person, and data analysis was done by two researchers independently.

### MBCT intervention

The MBCT program used in this study closely followed the 8-week MBCT manual developed by Segal et al. [23]. It comprised 8 weekly face-to-face group sessions, each lasting approximately 2 hours. The sessions included facilitator instruction, group practice and instructions for home practice. In brief, the manual followed a similar layout for each session and opened with introduction to a new theme (see Additional file 2 for themes), followed by short opening meditation and discussion. The group was then introduced to a new practice/exercise, which was followed by reflection, then review and instruction for at-home practice and followed by sitting meditation. A sample list of activities for session 1 is presented in Additional file 3.

The type of practices used in the MBCT curriculum are a combination of formal exercises/meditations such as body scan, sitting and walking meditation and mindful stretching; cognitive behavioural exercises and informal practices and discussions with personal reflections of everyday life events. A sample audio file of one of the meditations is available in Additional file 4.

Part of the intervention involved home practice assignments aimed at reinforcing the in-group learned techniques and strategies. The recommended home practice was up to 45 minutes a day for 6 days a week, with guided audio CD and outlined instructions for the home practice. The hand-outs and audio CD’s used for home practice are ready available from the published books respectively [23, 40].

To further reinforce the learned practices, the manual suggests that an additional full day of mindful practice take place between weeks 6 and 7 (usually on a weekend). In the full day of practice participants go through all the learned meditations one after another in silence, with the group reflection and discussion taking place at the end of the practice day. Due to resource constraint, in this study, the full day of practice was offered to participants after they have completed the 8-week course.

The program was delivered by two experienced MBCT practitioners who have been briefed on the key concerns and difficulties that IBD patients experience, as well as on the nature of the disease. Both practitioners had completed an 8-week MBCT course, maintained a personal practice and had facilitated a number of 8-week MBCT programs each over the previous five years, fulfilling the good practice guidance for teaching mindfulness-based courses [41].

Each weekly session was audio recorded except the last one due to failure of the recording device.

### Wait-list control

The control group continued to receive their standard care and in addition they received a leaflet entitled ‘Staying well with IBD’. The leaflet is readily available to download from the Crohn’s and Colitis UK website, but participants in the study received a printed copy [42]. After the 6-month follow-up data were collected, the wait-list group had the opportunity to attend a MBCT program.

### Data collection, assessments and outcomes measures

As this was an efficacy trial with the primary objective being to pilot the MBCT program with IBD patients and to assess the feasibility of the program and methodology in a definitive RCT, data were collected to assess trial feasibility [43]. Data were also collected on the proposed outcome measures to be tested in a definitive RCT.

#### Feasibility criteria and measures

The guidance for a good pilot study suggests setting a predetermined criterion for measuring the success of feasibility [44]. While literature suggests various figures [45, 46], the feasibility criterion for assessing success of feasibility in this study was set to at least 10% recruitment rate.

**Fig. 1:**
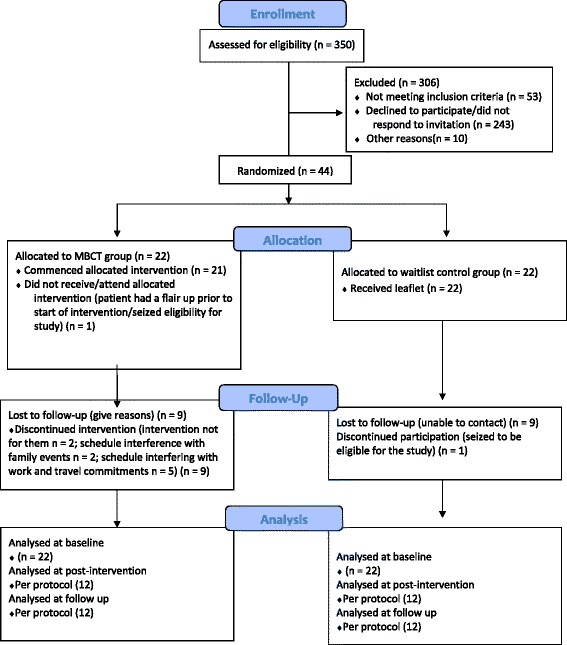
Consort diagram describing flow of patients through study

Screening and recruitment data were collected by the lead researcher on all patients considered for the study. Information was also collected on patients excluded with reasons for exclusion at each stage, date of recruitment and randomisation. A full CONSORT diagram [47] of subject flow is presented in Fig. 1 and a CONSORT checklist is available in Additional file 5.

To assess treatment compliance and to inform the estimated attrition rates for a full trial, facilitators recorded a weekly attendance log for each participant.

#### Baseline characteristics

Demographic data (age, sex, marital status, education and income) was obtained to assess the success of randomisation [48]. Data were also collected on participants locality (rural or urban), to assess if there is any relationship between rurality and drop-out rates.

#### Proposed outcomes measures

The following proposed outcomes were assessed at baseline, post intervention and at 6 months: depression, anxiety, dispositional mindfulness, disease activity and quality of life.

#### Depression

Low mood and depression symptoms was measured with the Beck’s depression inventory (BDI-II) [49]. The BDI-II is an established self-reporting tool for screening depression, and it consists of 21 group of statements, where the participant rates each statement on a four-point scale of severity. The statements refer to the last 2 weeks. The interpretation is based on a 0 to 63 total score, with higher total scores indicating more severe depressive symptoms. Previous studies indicate high test-retest reliability as well as high internal consistency [50, 51].

#### Anxiety

Anxiety was measured by the State and Trait Anxiety Inventory (STAI). STAI is a widely used self-reporting tool consisting of two parts: STAI- Y1 and STAI-Y2. Both parts consist of a 20-item scale, with STAI- Y1 measuring the state or current anxiety (anxiety related to an event) and STAI-Y2 measuring the trait or chronic anxiety (anxiety level as a personal characteristic). Participants are asked to rate each individual statement on a four-point scale, depending on how well each statement is describing the participants mood. The rating options are ranging from “not at all” to “very much so”. Each of the two parts of the STAI scores range between 20 and 80, with higher scores being positively correlated with higher levels of anxiety [52]. This tool is widely used to measure anxiety and regarded as highly reliable [8], but is particularly useful for IBD patients as each of the statements is focused on the cognitive symptoms of anxiety rather than mixing it with the somatic symptoms related to the disease.

#### Dispositional mindfulness

Dispositional mindfulness or mindful attention was measured using the Mindful Attention Awareness Scale (MAAS). This scale consists of 15 items that measure the frequency of which participants experience mindful awareness and mindful attention on a six-point Likert scale. The scale items refer to statements about everyday experiences graded by participants, using a scale of 1 to 6, which indicates how often each experience occurs. Higher scores reflect higher levels of dispositional mindfulness. The validation of this tool has been examined in a series of studies indicating strong psychometric properties and validity [53, 54].

#### Disease activity

Disease activity was assessed with an eight-item questionnaire Crohn’s Disease Activity Index (CDAI) for Crohn’s patients [55, 56] and six-item questionnaire Simple Clinical Colitis Activity Index (SCCAI) for ulcerative colitis patients. The CDAI has been validated prospectively and is the gold standard for the evaluation of CD disease activity [57, 58]. A decrease in the CDAI of 70 or 100 points has been defined as a CDAI-70 and CDAI-100 clinical response, respectively [59]. The SCCAI, as well as the CDAI, is a subjective disease activity index and rates overall well-being, daytime and night-time bowel movements, bowel movement urgency and rectal bleeding. SCCAI disease activity scores ≥3 correlate with active disease [60].

#### Quality of life

All participants were required to complete a disease-specific IBD quality of life (IBDQ) questionnaire. Although the questionnaire used in this study closely followed the validated IBDQ 32-item questionnaire that measures health-related QoL in IBD patients, the questionnaire was modified from the original version of the IBDQ. What remained were the same the four domains, including bowel symptoms, systemic symptoms, emotional factors and social factors. The only difference was that participants rated their symptom experience over the previous 2 weeks on a four-point Likert scale ranging from 0 (worse health) to 3 (best health) rather than 7-point. This was to reduce the burden of patients. Thus, scores ranged from 0 to 96 rather than 32 to 224 in the original IBDQ, and similarly, low scores indicated more severe disease activity and/or higher emotional and social dysfunction. IBDQ directly assesses the participant’s view on her/his disease and a relatively good correlation between the IBDQ and a widely used measure of disease activity, the Crohn’s Disease Activity Index is reported [61, 62].

### Sample size

Due to the nature of this pilot study, a formal sample size calculation was not performed. The determined sample size of *n* = 40 was calculated based on the estimated number of participants expected to complete the 8-week program. Full information on how n = 40 was achieved is reported elsewhere [37].

### Analysis

In line with the good practice guidance for analysis of any pilot study, the primary analysis of the study was descriptive [63, 64]. Descriptive data were calculated representing frequencies, means and standard deviations for all continuous data and n (%) for categorical data.

Further analysis was done to determine initial estimates of the parameters for the proposed outcome measures, for example, the mean and standard deviation required for sample size calculation for a future large RCT [65]. As this was an efficacy trial, the type of analysis was as per protocol [43]. For this, analysis of mixed covariance (ANOVA) statistical method was used. This method looked at the changes in outcome scores over time in the two different groups. A significance level of 5 % was chosen for proposed hypothesis testing. Data analysis was conducted using IBM SPSS version 19 software.

## Results

### Objective one: trial methodology

#### Eligibility, recruitment and dropout rates

Recruitment, intervention delivery and follow-up took place between May 2013 and March 2014. A total of 350 consecutive patients were assessed for eligibility. Study invitations were sent to 297 eligible patients and 44 consented to participate, giving a recruitment rate of 15 %. A consort diagram of patient flow is presented in Fig. 1. Although a total of 243 patients did not respond to study invitation, a recruitment target of 40 was achieved. In total, 44 participants were randomised, with 22 in each arm. Table 1 summarises reasons for discontinuing MBCT, how many sessions each of the participants who dropped-out attended and if they were from a rural or urban area. One participant randomised to the intervention arm ceased eligibility (had a flare-up) before the commencement of intervention, and one participant randomised to the wait-list control arm ceased eligibility (attended mindfulness course elsewhere while in the control) after randomisation. Data for another 18 participants were lost to follow-up (9 in each arm). In addition, 95.5 % of participants were recruited from one board only.

**Table 1:** Reasons for discontinuing mindfulness-based cognitive therapy (MBCT)

Reason	Number of participants (%)	Number of sessions attended	From rural area (%)
Not for them	2 (9)	1	1 (4.5)
Travel time	2 (9)	1	2 (9)
Family illness/carer	1 (4.5)	1	1 (4.5)
Work schedule interference	2 (9)	1	0 (0)
Family illness/carer	1 (4.5)	3	0
Acquired unrelated sickness	1 (4.5)	3	1 (4.5)
Seized eligibility before start of intervention	1 (4.5)	0	0 (0)
Total	10 (45)	10	5 (22.7)

#### Protocol adherence and success of data collection strategy

Completion of data collection at each time point is summarised in Table 2. This also indicates the degree of adherence to the research protocol. A log of attendance revealed that a total of 12 participants (56 %) completed at least four sessions from the intervention over the 8 weeks (Table 3). An overall of 24 participants (56 %) completed all assessments at post-intervention and 6-month follow-up.

**Table 2:** Data completion for research outcomes

	Control	MBCT	Total	Missing/Invalid
Total consented and randomised	22	22	44	n/a
Dropped out				
Prior start of MBCT	0	1	1	n/a
Prior to MBCT completion	10	9	19	n/a
Prior to 6 month follow up	0	0	0	n/a
Baseline BDI-II	22	21	43	1
Post-MBCT completion BDI-II	12	12	24	20
6-month follow-up BDI-II	12	12	24	20
Baseline STAI-Y1	21	20	41	3
Post-MBCT completion STAI-Y1	12	12	24	20
6-month follow-up STAI-Y1	12	12	24	20
Baseline STAI-Y2	22	20	42	2
Post-MBCT completion STAI-Y2	12	12	24	20
6 month follow up STAI-Y2	12	12	24	20
Baseline MAAS	22	22	44	0
Post MBCT completion MAAS	12	12	24	20
6-month follow-up MAAS	12	12	24	20
Baseline IBDQ	21	21	42	2
Post-MBCT completion IBDQ	12	12	24	20
6-month follow-up IBDQ	12	12	24	20
Baseline DA	21	22	23	1
Post-MBCT completion DA	12	12	24	20
6-month follow-up DA	12	12	24	20

*Abbreviations*: *BDI-II* Beck Depression Inventory II, *DA* Disease Activity, *IBDQ* Inflammatory Bowel Disease Questionnaire, *MAAS* Mindfulness Attention Awareness Scale, *MBCT* Mindfulness Based Cognitive Therapy, *STAI-Y1* and *Y2-State* Trait Anxiety Inventory

**Table 3:** Mindfulness-based cognitive therapy (MBCT) attendance log

	Week 1	Week 2	Week 3	Week 4	Week 5	Week 6	Week 7	Week 8	Sessions Missed	Sessions attended
1.						x			1	7
2.					x	x	x		3	5
3.			x		x	x		x	4	4
4.		x	x	x	x	x	x	x	7	1
5.								x	1	7
6.									0	8
7.		x	x	x	x	x	x	x	7	1
8.				x	x	x	x	x	5	3
9.									0	8
10.									0	8
11.			x		x	x	x	x	5	3
12.		x	x	x	x	x	x	x	7	1
13.							x		1	7
14.									0	8
15.									0	8
16.	x	x	x	x	x	x	x	x	8	0
17.	x		x	x	x	x	x	x	7	1
18.									0	8
19.		x	x	x	x	x	x	x	7	1
20.		x	x	x	x	x	x	x	7	1
21.		x	x	x	x	x	x	x	7	1
22.									0	8

X-missed a session

#### Baseline characteristics

Table 4 shows the age, sex, income, disease type, marital status and education distribution for the 44 consented participants. An independent T-test between groups at baseline showed no significant differences between the two arms for baseline characteristics.

**Table 4:** Baseline characteristics

	Intervention	Control
Age (years) (n, mean (sd))	22, 48.59 (12.046)	22, 49.68 (15.370)
Sex (F (n,%), M(n,%))	16,(72.7) 6,(27.3)	18,(81.8) 4,(18.2)
Income (n, %)		
less 10K	1 (4.5)	6 (27.3)
10K-19K	7 (31.8)	6 (27.3)
20K-29K	4 (18.2)	2 (9.1)
30K-39K	4 (18.2)	4 (18.2)
40K-50K	2 (9.1)	3 (13.6)
50K+	4 (18.2)	1 (4.5)
Disease type		
CD (n, %)	9 (40.9)	12(54.5)
UC (n, %)	13(59.1)	10(45.5)
Marital status		
Single (n, %)	5 (18.2)	9 (40.9)
Married/cohabiting (n, %)	15 (68.2)	8 (36.4)
Widowed (n, %)	0 (0)	3 (13.6)
Separated/divorced (n, %)	3 (13.6)	1 (4.5)
Education (High school n, %)	9 (40.9)	9 (40.9)
(Diploma n, %)	9 (40.9)	7 (31.8)
(Degree or above n, %)	4 (18.2)	5 (27.2)

*CD* Crohn's Disease, *F* female, *M* male, *N* number, *SD* standard deviation, *UC* Ulcerative colitis

### Objective two: initial estimates on parameters of the proposed outcome measures (depression, anxiety, dispositional mindfulness, disease activity and quality of life)

A mixed ANOVA was conducted on all dependent variables: depression, anxiety, dispositional mindfulness, disease activity and quality of life. All assumptions with regard to outliers, normal distribution, homogeneity of variances and co-variances and sphericity were tested and met. The means and standard deviation (S.D.) of all proposed outcomes for MBCT and the wait-list group over the three time points are presented in Table 5.

**Table 5:** Means and standard deviation of proposed outcomes at baseline, post-MBCT and follow-up

Measured outcome	Condition	Baseline			Post-MBCT			6 month Follow up		
		Mean	S.D.	N	Mean	S.D.	N	Mean	S.D.	N
BDI-II	MBCT	14.36	9.520	22	10.67	13.996	12	13.75	16.355	12
	Wait-list	15.57	7.291	21	14.23	10.158	12	14.17	9.173	12
STAIY1	MBCT	38.76	11.397	21	37.74	15.635	12	39.67	16.183	12
	Wait-list	37.26	10.429	20	43.67	5.806	12	45.16	9.347	12
STAIY2	MBCT	45.50	10.318	22	41.67	16.396	12	42.58	16.368	12
	Wait-list	47.45	8.666	20	47.08	6.431	12	45.92	7.354	12
MAAS	MBCT	3.6586	.7935	22	4.2758	1.0342	12	4.1450	1.1675	12
	Wait-list	3.4005	.6655	22	3.5769	.5802	12	3.5433	.7867	12
CDAI	MBCT	101.56	60.28052	3	69.0000	86.15683	3	18.6833	15.01002	3
	Wait-list	145.866	84.76086	8	101.8750	66.69212	8	139.7150	83.44660	8
SCAI	MBCT	4.3750	2.77424	8	3.8750	3.39905	8	5.5000	4.17475	8
	Wait-list	2.2500	2.62996	4	4.5000	3.0000	4	5.0000	4.39697	4
IBDQ	MBCT	34.3333	12.17922	21	31.0833	18.0225	12	34.8333	23.7863	12
	Wait-list	36.5714	14.40684	21	33.9167	15.1864	12	36.8333	12.0667	12

Depression (BDI-II); State anxiety (STAIY1); Trait anxiety (STAIY2); Dispositional mindfulness (MAAS); Crohn’s disease activity index (CDAI)

Ulcerative colitis activity index (SCAI); Disease specific QoL (IBDQ)

#### (BDI-II) depression

Per-protocol analysis revealed an improvement in depression scores in the MBCT group at post-intervention and follow-up. There was a statistically significant interaction between the MBCT group and time on depression scores (F(4,84) = 3,975, *P* = .027, partial η2 = .173).

#### (STAIY1) state anxiety

There was an improvement in state anxiety score in the intervention arm over the post-intervention and follow up period; however, the difference between the arms over time was not statistically significant (F(4,84) = 2,809, *P* = .083, partial η2 = .135).

#### (STAIY2) trait anxiety

When the per-protocol population was analysed, the trait anxiety scores between the two arms showed a statistically significant interaction between the MBCT group and the time on trait anxiety scores (F(4,84) = 3,286, *P* = .048, partial η2 = .147).

#### MAAS (dispositional mindfulness)

Dispositional mindfulness scores showed an improvement in the MBCT arm in comparison to the wait-list, with a statistically significant interaction between the MBCT group and time on dispositional mindfulness (F(4,84) = 3,998, *P* < =.034, partial η2 = .174).

#### DA (disease activity)

Although disease activity showed improvement in the MBCT arm, there was no statistically significant interaction between the MBCT and time on Crohn’s disease activity scores (F(4,84) = 1,410, *P* = .277, partial η2 = .168) or between the MBCT group and time on ulcerative colitis activity scores (F(4,84) = 2,927, *P* = .083, partial η2 = .268).

#### IBDQ (IBD quality of life)

While there was a small improvement in the IBDQ score at the 6-month follow-up, there was no statistically significant interaction between the MBCT group and time on quality of life scores (F(4,84) = .845, p = .437, partial η2 = .043).

### Objective three: sample size calculation

The sample size for a full RCT is calculated on the basis of the proposed primary hypothesis and clinically meaningful effect sizes of changes in depression scores BDI. A change of five scores in BDI is deemed to be clinically meaningful [66]. We have based our sample size estimate on the most conservative standard deviation of 11.89. To detect a mean difference in BDI score of five points at week 8 with a two-sided significance level of 5 % and power of 80 % with equal allocation to two arms would require 90 patients in each arm of the trial. To consider and allow a 44 % drop-out (finding from this study), then 129 IBD patients should be recruited per arm (258 in total).

## Discussion

This paper describes a pilot randomised control trial of Mindfulness-based Cognitive Therapy for IBD. The results showed that it would be feasible to conduct a full RCT. In conducting this investigation, we have identified areas of critical importance if a subsequent study of MBCT for IBD is to going to be conducted. These areas are related to recruitment and retention, data collection and trial design as well as to the intervention.

### Objective one: trial methodology

#### Consent, recruitment and retention

##### Consent and baseline

Recruitment for this trial was difficult, even though the recruitment target was reached. It was estimated that each individual appointment for discussing consent and making baseline assessments would last approximately 45 minutes, but in reality lasted approximately 1.5 hours. This was not due to the assessments taking longer, but due to fact that for most of the participants this was an opportunity for them to voice their difficulties, particularly the stress and anxiety related with the condition. Because much of the information was shared before actually signing the consent form, this information was not captured for analysis. In addition, tester sessions were offered to prospective participants before they made a decision to participate. This idea came from some of the prospective participants wanting to ‘test drive’ the intervention. Although only three signed up for the tester session, all three participants that came for a tester session decided to participate and completed all the trial assessments.

##### Recruitment

Although the recruitment was conducted in two NHS Boards (approximately 2,341 IBD patients), 95.5 % of participants were recruited from one of the boards. A number of strategies were devised to maximise the number of patients screened for eligibility into the study. The most effective strategy of recruitment (93.2 %) was through a letter of invitation send by an IBD specialist nurse. We believe there are at least two possible explanations regarding this. Firstly, it is possible that this strategy was most effective due to the fact that there was already an established trusted relationship between the patient and the IBD nurse, and the response to the invitation to participate reflected that trust. And secondly, it could be the actual high number of letters that were sent to patients could be the reason for the good response rate. Nonetheless, this strategy was a lengthy process of going through IBD patient records and sending invitation and information packs every week for few months. To improve future recruitment, literature suggests three key areas of relevance: infrastructure, professional and public engagement with research, and methodological innovation [67]. A dedicated recruitment person working closely with the IBD nurse, using current networks such as Crohn’s and Colitis UK to aid recruitment or offering incentives for prospective participants could make this process more effective. These suggestions should be considered if a full RCT is to be conducted.

##### Retention

Both arms experienced an equally high dropout rate of 44 %, with 33 % attending only one session in the intervention arm. If we only look at the intervention arm, the high drop-out rate suggests that the intervention may not be suitable or acceptable to all IBD patients. However, whereas seven participants (33 %) attended only one session, only two of them said the intervention was not for them. The reasons that the other five participants gave were that they realised the travel time commitment was too much (2), there was work schedule interference (2) and there was a family illness (1). Although at recruitment, the commitment required for the course was particularly highlighted, it appears that the participants either overestimated their other commitments and travel time or were overenthusiastic to start with and had a decline in motivation or had a change of circumstances by the time the intervention started. The last one is a possibility if taken into consideration that the time between recruitment and start of intervention was around 5 months for some participants [68] and should be addressed in any future trial.

If we look at the wait-list arm, the dropout rate was the same, with nine participants not responding to the two communication attempts to complete assessments after the post-intervention period or follow-up. In this arm, it is possible that the participants lacked the motivation to stay in the trial and perhaps declined participation outside of the trial setting, which could also be due to the long wait between baseline assessment and post-intervention and follow-up assessment. The other possibility is that perhaps the participants in the control were disappointed that they were not selected to be in the intervention arm, although they were offered the intervention after all data were collected. This was at least the case with the one participant that was excluded from the trial after they breached the protocol and did the intervention while in the control group. In addition, a log was kept for the wait-list arm attending the intervention after the completed assessments, and their attendance was close to 100 %, with no drop-outs. Participants in the wait-list arm remained motivated to complete all the follow-up assessments because they knew they would be eligible for the intervention after all data had been collected. In summary, while high drop-out rates are a recognised occurrence in psychological intervention trials [69–71], the demand for psychological interventions in IBD is pertinent, and judging from those that attended the intervention, it appears that careful patient selection remains essential [72, 73].

#### Protocol adherence and success of data collection strategy

Complex psychological interventions are by definition difficult to standardise and measure, and this always should be considered [74]. Whereas a log of attendance was kept for the intervention arm in this study, it was very difficult to assess how much home practice the participants did, and home practice was a particularly vital component of the intervention. To be able to assess the time spent on home practice and truly assess the effectiveness of the intervention in the future, a measurable log of home practice might be introduced.

All data collected in the trial, including the consent form, were collected by the lead researcher. This is particularly important to support a robust methodology, especially when front line clinicians’ priorities and time is constrained. However, there is a debate that involving clinicians in data collection is important, particularly in context of culture and demonstrating the concept of working together [75], and perhaps this should be considered in a future trial.

Data were collected at three time points, with the last one at 6 months. The six-month follow-up was to assess the mechanism of how feasible it is to collect follow-up data for a full RCT, thereby assessing the sustainability of any effectiveness of the intervention. Ideally, a longer follow-up, such as 12 months, would give us better information about any sustainable changes in a full RCT. Initially, the pilot considered testing the feasibility of data collection at 12 months; however, there were few points taken into consideration. The recruitment process was stretched over a few months, and judging by the dropout rate in both arms and literature [76], it could have contributed towards further drop-outs in both arms, predominantly in the wait-list arm if patients had to wait even longer to be eligible to attend MBCT after all follow-up assessments. In a future trial, this could be overcome by having a designated research person working fulltime on recruitment, which could reduce the recruitment time, thereby enable a shorter lag time between recruitment and the start of intervention.

#### Objective two: initial estimate on parameters of the proposed outcome measures (depression, anxiety, dispositional mindfulness, disease activity and quality of life)

With respect to the initial calculations on parameters for the proposed outcome measures for a full RCT, all outcome measures in the present study were validated and found to be reliable measures. Although this analysis has its limitations due to the small sample size and should not be generalised, it provides encouragement that MBCT has the potential to help with management of overall symptoms for IBD patients.

We measured the dependent variables (proposed outcome measures) over time in the two different groups and wanted to assess whether the dependent variables responded differently over time in the groups. Thus, a mixed ANOVA (with both between-subjects and within-subject factors) analysis was conducted. For this analysis, we looked at the group*time interaction where the group (MBCT or wait-list) was the between factor, and time (baseline, post-intervention and follow-up) was the within factor [65]. We did the analysis for each of the dependent variables: depression, anxiety, dispositional mindfulness, disease activity and quality of life. This also gave the initial estimates of the parameters to the proposed outcome measures (mean and standard deviation) required for a sample-size calculation for a future large RCT [64].

The mixed ANOVA per protocol analysis showed a statistically significant interaction between the group (MBCT and wait-list) and time (baseline, post-intervention and follow-up) on depression (BDI-II), trait anxiety (STAI-Y2) and dispositional mindfulness (MAAS) scores. This is particularly interesting as the literature suggests that high depression and anxiety are closely linked with neuroticism. In addition, the most common personality trait in IBD patients is reported to be neuroticism [77–80]. High neuroticism scores are related to reduced psychosocial wellbeing, psychological adjustment and quality of life in patients with IBD [81] or higher depression and anxiety vulnerability. Although we did not directly measure neuroticism scores, their relation to depression, state anxiety and dispositional mindfulness has been well reported.

Considering that more than 30 % of IBD patients report suffering from depression and that the preliminary analysis showed that depression scores in the MBCT group improved over time when compared to the control, these results are very promising. Further to this, MBCT had significant effect on trait anxiety, whereas the effect on state anxiety was not significant. This is particularly interesting because changes in the trait anxiety scores suggest that they are not temporary changes but they are more sustainable comparing to the state anxiety. For example, a person who has a high trait anxiety, views typical daily situation as more threatening than those with lower trait anxiety and so responds with a higher state anxiety. High trait anxiety is often linked with neuroticism and higher vulnerability to depression [82]. Reducing the trait anxiety could in return lower the vulnerability for depression. In the long run, and taking into account that IBD is a lifelong condition with distressing symptoms, the potential of MBCT to help IBD patients to respond to daily situations, as well as the disease symptoms in a less stressful way, is certainly promising. Of course, to get a better idea of how sustainable this is, a full RCT has to be conducted.

The other interesting finding was the improvement in dispositional mindfulness scores. Dispositional mindfulness has been shown to moderate the relationship between neuroticism and depressive symptoms [83]. A study suggests that neuroticism is significantly related to depression in those with low to medium levels of dispositional mindfulness but not in those with relatively high levels of mindfulness. It also suggests that increased dispositional mindfulness may act as a protective factor against the effects of negative emotional reactivity by neuroticism. This could be very important for the future tailoring of treatment based on patient characteristics which is a well-accepted approach in IBD management (for example, pharmacotherapy).

Disease activity, state anxiety and quality of life, showed improvement over a period of time, but statistical significance was not detected. This could be due to the small numbers, particularly with the disease activity, where the subgroup of CD and UC were very small to compare between the two arms. The biggest surprise is that quality of life change was very small and, in fact, did not mimic the change in depression scores or trait anxiety, as had been expected. One of the possible explanations is that the sample was too small to detect any significance. The second reason could be that the questionnaire used was an adapted version of the validated IBDQ questionnaire and was not sensitive enough to detect any real changes. What is also very interesting is that in both arms, MBCT and the wait-list, there was a reduction in the quality of life scores at post-intervention, and then increase of scores at the 6-month follow-up. It is unclear whether any external factors contributed to this or to the coincidental worsening in the quality of life for both arms at post-intervention, with improvement at the 6-month follow-up.

### Objective three: sample-size calculation

We estimated a sample-size calculation for a future trial based on the dropout rate of this trial. However, we should consider that the estimate from this study is only an indication to what the ‘true’ dropout rate is, and perhaps, the consideration of estimates from other trials with similar type of intervention for this patient group should be not dismissed.

## Conclusions

We completed an exploratory pilot RCT despite challenges in recruitment. Based on the study findings and the experience of conducting the pilot trial, we would recommend a definitive multicentre trial with 129 participants in each arm. Whereas the recommendations for consent, randomisation and data collection are to be conducted by a dedicated research team, recruitment should be in collaboration with clinical staff, particularly IBD specialist nurses, to maximise recruitment. Although dropout rates were higher than expected, a future trial could minimise this by decreasing the time lag between recruitment and start of intervention. Short tester sessions could be offered to all potential participants to help with appropriate patient selection and improving retention. Retention rates were the same in both arms, which suggests that randomisation was successful. A measurable log of home practice should be introduced to better assess protocol adherence and intervention compliance and therefore determine the ‘true’ effectiveness of the intervention. Information on medication or dosage changes during the study period should be collected to assess if it affects outcomes. The improvement in depression, trait anxiety and dispositional mindfulness scores in the intervention arm at post-intervention and follow-up suggest that MBCT holds a potential to improve overall symptom management and quality of life for IBD patients.

## Additional files

Additional file 1:
**Inclusion and exclusion criteria.** (DOCX 19 kb) Additional file 2:
**Weekly session themes.** (DOCX 15 kb) Additional file 3:
**A sample list of activities for session 1.** (DOCX 13 kb) Additional file 4:
**Three-minute breathing space Audio.** (ZIP 2972 kb) Additional file 5:
**CONSORT check list.** (DOC 217 kb)

## Abbreviations

BDI-II: Becks Depression Inventory
CD: Crohn’s disease
CDAI: Crohn’s Disease Activity Index
IBD: inflammatory bowel disease
IBDQ: Inflammatory Bowel Disease Quality of Life
MAAS: Mindful Attention Awareness Scale
MBCT: Mindfulness-based Cognitive Therapy
MRC: Medical Research Council
QoL: Quality of Life
RCT: randomised controlled trial
SCCAI: Simple Clinical Colitis Activity Index
STAI: State and Trait Anxiety Inventory
UC: ulcerative colitis
