# Supplementary material for: Mindfulness-based cognitive therapy for inflammatory bowel disease patients: findings from an exploratory pilot randomised controlled trial
Source: Trials. 2015 Aug 25;16:379. doi: 10.1186/s13063-015-0909-5 (PMC4549082; doi:10.1186/s13063-015-0909-5)
Supplement: Additional file 1: — Inclusion and exclusion criteria. (DOCX 19 kb) [file 13063_2015_909_MOESM1_ESM.docx]

**Inclusion criteria**

1. Be able to verbally communicate and write in English (English does not have to be their first language).

2. Able to give informed consent.

3. Age of 18 years or over (no upper limit).

4. Confirmed diagnosis of Crohn’s disease or ulcerative colitis (by clinician).

5. Ability to do light exercise (for example, to lift arms above the head or bend knees) because part of the practices in the program require this movement.

6. Able to commit to attend the eight sessions (participants should consider their personal circumstances to assess if this is practical and feasible for them).

7. To be able to commit to do home practice of up to 45 minutes daily over the 8 weeks of the study (this is a core component of the program).

8. No change of antidepressants (dose or type) within the last 3 months. Any change of antidepressants within the last 3 months might interfere with the program.

9. Participants will have to be in remission of symptoms.

**Exclusion criteria**

Anyone not meeting the above criteria by definition will be excluded from the study. In addition, the following exclusion criteria will apply:

1. Major psychiatric illness. The treatment for a major psychiatric illness may interfere with the program.

2. Active alcohol or drug dependency. Any alcohol or drug dependency may interfere with the program.

3. Scheduled for major surgery in the next 3 months. Any scheduled surgery within the next 3 months will interfere with the program schedule.

4. Participation in a pharmacological study or psychological intervention study within the last 6 months or intention to participate in a pharmacological study during the duration of this study. Both will interfere with the program.

5. Have recently (within the last 3 months) been prescribed antidepressants. Any change of antidepressant in the last 3 months may interfere with the program.

6. With exacerbated symptoms (acute phase). Having exacerbated symptoms will make it very difficult for participants to attend the two hourly sessions or to commit to the home practice. This could cause extra unwanted stress for the patient.
