# Supplementary material for: Mindfulness-based cognitive therapy for inflammatory bowel disease patients: findings from an exploratory pilot randomised controlled trial
Source: Trials. 2015 Aug 25;16:379. doi: 10.1186/s13063-015-0909-5 (PMC4549082; doi:10.1186/s13063-015-0909-5)
Supplement: Additional file 2: — Weekly session themes. (DOCX 15 kb) [file 13063_2015_909_MOESM2_ESM.docx]

Week 1: Awareness and automatic pilot

Week 2: Living in Our Heads

Week 3: Gathering the Scattered Mind

Week 4: Recognizing Aversion

Week 5: Allowing/Letting Be

Week 6: Thoughts Are Not Facts

Week 7: How Can I Best Take Care of Myself?

Week 8: Maintaining and Extending New Learning
