# Supplementary material for: Mindfulness-based cognitive therapy for inflammatory bowel disease patients: findings from an exploratory pilot randomised controlled trial
Source: Trials. 2015 Aug 25;16:379. doi: 10.1186/s13063-015-0909-5 (PMC4549082; doi:10.1186/s13063-015-0909-5)
Supplement: Additional file 3: — A sample list of activities for session 1. (DOCX 13 kb) [file 13063_2015_909_MOESM3_ESM.docx]

**A sample list of activities for session 1**

Theme: Awareness and automatic pilot

1. Establish the orientation of the class

2. Set ground rules regarding confidentiality and privacy

3. Ask participants to pair up and introduce themselves to each other than to the group as a whole, giving their first names and if they wish, saying what they hope to get out of the program

4. The raisin exercise

5. Feedback and discussion of the raisin exercise

6. Body scan practice-starting with short breath focus

7. Feedback and discussion of body scan

8. Home practice assignment:

• Body scan for 6 out of 7 days

• Mindfulness of a routine activity

• Distribute audio files: cd’s for those that not have email and session 1 participant hand-outs.

9. Discuss in pairs:

• Timing for home practice

• What obstacles may arise

• How to deal with them

10.  End the class with a short 2–3 minute focus on the breath.
